# Growth in neonates with congenital kidney failure requiring continuous kidney replacement therapy

**DOI:** 10.1007/s00467-025-06887-y

**Published:** 2025-08-07

**Authors:** Kara Short, Janelle Hanick, Perrin Bickert, Jessica Potts, David Askenazi

**Affiliations:** 1 Children’s of Alabama, Birmingham, USA; 2 Pediatric and Infant Center for Acute Nephrology, Children’s of Alabama and University of Alabama at Birmingham, Birmingham, USA; 3 Division of Pediatric Nephrology, University of Alabama at Birmingham, Birmingham, USA

**Keywords:** Continuous kidney replacement therapy (CKRT), Congenital kidney failure (CKF), Neonate, Infant, Peritoneal dialysis, Nutrition

## Abstract

**Background:**

With advanced technology, survival of neonates with congenital kidney failure (CKF) requiring continuous kidney replacement therapy (CKRT) has improved. Nutrition is essential but difficult to attain as CKRT removes proteins and micronutrients, and many patients have multiple co-morbidities. Scant data exist to guide clinicians on appropriate energy requirements for growth.

**Methods:**

We performed a retrospective study of neonates with CKF admitted to Children’s of Alabama between 2016 and 2022 who required KRT within 10 days. We evaluated risk factors and growth in the 18/24 (75%) infants who survived to 90 days. Our primary and secondary outcomes were length z-score ≥  − 2 vs. <  − 2 at 90 days and weight z-score ≥  − 2 vs. <  − 2 at 90 days, respectively. Demographics, comorbidities, CKRT Dose Eras (1-body surface area (2000/1.73/m^2^/hr) vs. 2-weight-based era (24 ml/kg/hr)), and Nutrition Era 1 vs. 2 were evaluated.

**Results:**

At 90 days, 7/18 (38.9%) had length z-score ≥  − 2 while 10/18 (55.6%) had a weight z-score ≥  − 2. Factors for weight z-score ≥  − 2 include time to PD transition and CKRT Dose Era 2. Factors for length z-score ≥  − 2 included Era with higher calorie and protein goal targets (both *p* < 0.01).

**Conclusions:**

Malnutrition in neonates with CKF on CKRT is high. More studies are needed to better understand optimal strategies to ensure adequate growth. Until then, we recommend 24 ml/kg/hr clearance dose and prescribing at least 130 kcal/kg/day and 4 g/kg/day amino acids to target higher actual intake to start for these patients.

**Graphical abstract:**

**Figure:**
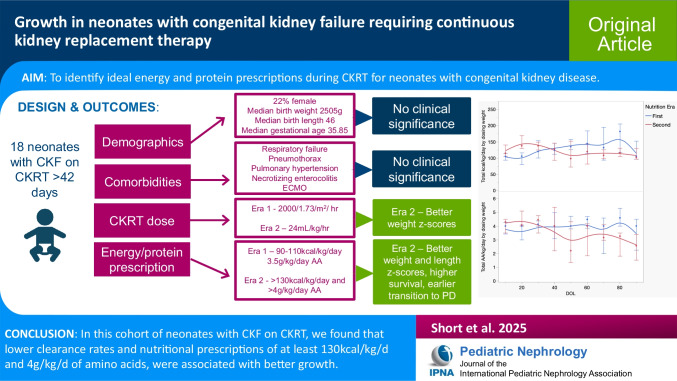
A higher resolution version of the Graphical abstract is available as Supplementary information

**Supplementary Information:**

The online version contains supplementary material available at 10.1007/s00467-025-06887-y.

## Introduction

With advancing technology, more neonates with congenital kidney failure (CKF) requiring continuous kidney replacement therapy (CKRT) are being given a chance at life [1]. The most common causes of CKF include congenital anomalies of the kidney and urinary tract (CAKUT) and genetic disorders including congenital nephrotic syndrome and polycystic kidney disease [2]. These neonates are at risk for malnutrition due to increased metabolic demands, removal of nutrients from dialysis, and intolerance of enteral nutrition [3]. There are many instances when these patients may not receive full nutrition, like while awaiting a PD catheter to heal, on surgical procedure days, and when complications limit the provision of full nutrition. Despite the advancements in technology and care for neonates with CKF over the last decade, little data exist to guide providers on prescribing appropriate nutrition to optimize growth.

As nearly one third of growth in childhood happens in the first 2 years of life, it is imperative that this population receives appropriate nutrition for the entirety of their hospital course [4]. Not only is nutrition essential for growth, but during critical illness as well, when patients suffer muscle protein breakdown and catabolism [5]. Furthermore, without steady growth, milestones in care (i.e., transition from manual to automatic peritoneal dialysis (PD)) cannot be achieved. The 2020 Pediatric Renal Nutrition Taskforce recommendations for children on dialysis provide consensus guidelines; however, they do not address the specific calorie and protein prescription requirements in context early after birth, especially when neonates, including preterm neonates, are receiving life-saving therapies for their critical illness. Also, as there are no current evidence-based recommendations for CKRT dosing in term and preterm neonates, recommendations for other modalities are difficult to translate to this population. Still, these recommendations suggest that energy and protein intake should approximate that of healthy children of the same age [6]. The Kidney Disease Outcomes Quality Initiative (KDOQI) recommends that while patients are on CKRT, higher protein intake is needed to address losses in dialysis [7].

A single-center study of eight neonates with CKF on CKRT showed that delayed height and growth velocity is very common. In fact, all eight of the reported patients did not meet goals until 6 months after initiation of CKRT while receiving a prescribed 90 ml/kg/day total fluid intake. Patient-specific calorie and protein prescriptions were not analyzed, but the authors note that, at times, nutritional volume and total protein increased once patients were placed on CKRT [8]. To improve our understanding of the nutritional needs for critically ill neonates with CKF who require CKRT, we performed a retrospective observational cohort study to investigate the nutritional, CKRT, and patient-related factors that are associated with growth in survivors with CKF on CKRT.

## Methods

### Population

This retrospective single-center observational cohort study includes neonates admitted to the Neonatal Intensive Care Unit (NICU) at Children’s of Alabama (COA) in Birmingham, AL, between January 1, 2016, and December 31, 2022, who met the following criteria: (1) began CKRT in the first 10 postnatal days, (2) required KRT for at least 42 days, and (3) survived to 90 days. We found that 18 patients met criteria (17 with congenital kidney failure and one with severe perinatal acute kidney injury which did not resolve). Data were extracted from the electronic medical record and the Pediatric and Infant Center for Acute Nephrology (PICAN) KRT quality improvement databases. The IRB at University of Alabama at Birmingham (UAB) approved this study with a waiver of parental/guardian informed consent (IRB – 300,002,149 – A Retrospective Analysis of Understanding Neonatal Dialysis Energy Requirements).

### Outcome measurements

The primary outcomes were the 90-day z-score ≥  − 2 for length and weight. Anthropometric measurements were obtained by the bedside NICU nurses and NICU dietitians. Weights were obtained twice daily according to our COA Neonatal CKRT guidelines. Dry weight estimation was determined collaboratively with the nephrology and neonatology clinical teams at least weekly, using several factors including vital signs and physical exam. Length measurements were collected weekly. Length and weight z-scores were calculated using sex-specific WHO growth charts for sex using corrected post-menstrual ages. For the data analysis, anthropometric measurements were captured at 10-day intervals up until 90 days and then at 30-day intervals until 180 days.

### Comparators—risk factors

Risk factors evaluated include demographics, postnatal day of initial KRT, days to initiation of PD, days on ventilator, extracorporeal membrane oxygenation (ECMO) requirement, surgeries, complications, co-morbidities, and days of CKRT. In addition, we evaluated how clinical practice modifications of CKRT prescribed dose and nutritional goals impacted growth. We evaluated two eras because our program’s CKRT prescribed dose changed in May 2018 from Dose Era 1 (body surface area approach of 2000 ml/1.73/m^2^/h) to Dose Era 2 (weight-based approach of 24 ml/kg/h across the institution). Similarly, we evaluated two additional eras related to CKRT nutritional recommendation changes that occurred from May 2019 from Nutrition Era 1 (90–110 kcal/kg/day and 3.5 amino acid g/kg/day) to Nutrition Era 2 (at least 130 kcal/kg/day and at least 4 g/kg/day).

### Dialysis modalities

All patients initially received CKRT as part of standard of care at our institution. We utilize CKRT via continuous veno-venous hemofiltration (CVVH) with Aquadex machine and UF500 filter (Nuwellis, Eden Prairie, MN), and/or continuous veno-venous hemodialysis (CVVHD) via in-line filter (Minntech 400, Medcomp, Minneapolis, MN) connected via shunt to extracorporeal membrane oxygenation (ECMO). Heparin, bivalirudin, and/or no anticoagulation is used for all patients, depending on risk for coagulopathies. Prismasol BGK2/3.5 (Baxter International, Deerfield, IL) is used for all modalities, with additives adjusted by the nephrology provider to maintain homeostasis. A peritoneal dialysis (PD) catheter is placed when the patient is stable enough to have an elective surgery, and use is delayed at least 2 weeks to allow the catheter to heal per Standardizing Care to Improve Outcomes in Pediatric ESKD (SCOPE): A National Quality Improvement Initiative: Peritoneal Dialysis Bundles. As patients grow and stabilize, the number of hours on CKRT decreases to allow more time for family bonding and occupational/physical therapies. Patients then transition to manual PD (Dialy-Nate, Utah Medical Products, Midvale, Utah) with continuous hourly cycles of 10 ml/kg/h, advancing as tolerated. Manual PD is transitioned to automated PD cycler (Home Choice Automated Peritoneal Dialysis System, Baxter, Deerfield, IL) for home therapy once the infant is large enough to tolerate the minimal fill volumes of 150 ml required of the machine with a goal Kt/V of 1.7 [9].

### Nutrition parameters

Parenteral and enteral nutrition were prescribed collaboratively by the neonatology and nephrology team members (physicians, nurse practitioners, and dietitians) based on in-center guideline recommendations created by the above team members. Nutrition was initiated as soon as able, primarily provided parenterally and transitioned to enteral nutrition by mouth or via enteral feeding tube (nasogastric, orogastric, transpyloric, gastrostomy tubes) as tolerated. All of the cohort received parenteral nutrition, as well as enteral feeds via a feeding tube at some point in their hospitalization. Medications, type of formula, concentration of enteral feeds, electrolyte and base supplements, protein, and calories were adjusted frequently by the clinical team to meet growth targets and laboratory homeostasis. Enteral nutrition recommendations begin with breast milk and, if not sufficient or available, the addition of the infant kidney formula (Similac 60/40; Abbott Global) at 20 kcal/oz. Protein and calorie modulators were only used when the patient transitioned to PD if necessary to meet nutrition goals and fluid restrictions sometimes needed on PD.

When patients transitioned to PD, their nutrition goals changed. Calories from glucose absorption through peritoneal fluids were incorporated into the daily nutritional calorie prescription. Due to this additional glucose exposure, a baseline of 90 kcal/kg/day and 2 g/kg/day amino acids was recommended by our nephrology team to start. Water-soluble vitamins were delivered parenterally in TPN and dosed by weight, or enterally via Nephronex (CWI Medical, Edgewood, NY) if tolerated.

After the initial prescription goals were achieved, the clinical team adjusted calories based on dry weight gain targets of 15–20 g/day for premature infants < 2 kg, preemies > 2 kg 20–30 g/day, and for term infants 0–4 month, 23–34 g/day was used based on the Pediatric Nutrition Reference Guide compiled by Texas Children’s and the WHO Child Growth Standards [10]. Protein intake was adjusted to keep blood urea nitrogen (BUN) levels between 40 and 60 mg/dl. Changes were made by increasing amino acids by 0.5–1 g/kg/day every 3 days if below 40 mg/dl, or by decreasing amino acids by 0.5–1 g/kg/day every 3 days until BUN was < 60. Nutrition and electrolytes were reviewed daily by the nephrology providers and nephrology RD, and suggested changes were then discussed with the neonatal team.

### Evaluation of average number of calories and protein prescribed

To evaluate the calories prescribed for this cohort, we recorded the nutrition prescription found in the electronic medical record (EMR) every 10 days of life for the first 90 days. We then analyzed the median prescribed caloric (kcal/kg/day) and protein (g/kg/day) intake for each infant both enterally and parenterally. Actual calories and protein consumed by the patient were not assessed.

### Statistical analysis

Weight and length z-score were calculated using the World Health Organization (WHO) growth chart corrected for gestational age. Categorical data were reported as *N* (%), and differences between groups were evaluated by the *X*^2^ test. If any cell had < 5, we used Fisher’s exact test. Continuous data were reported as median (IQR), and differences between groups were tested using the Mann–Whitney *U* test. A *p* value < 0.05 was considered statistically significant.

## Results

### Demographics

Between January 2016 and December 2022, 59 neonates received CKRT in our NICU at Children’s of Alabama. The demographics of the 18 survivors to 90 days are shown in Tables 1 and 2. Specifically, 77.8% were male, the median (IQR) gestational age (GA) was 35.9 (34.5, 37.0) weeks, birth weight was 2505 (2252, 2845) g, birth weight z-score was 0.45 (− 0.28, 1.18), and birth length z-score was − 0.24 (− 1.1, 0.5). Median ventilator days were 48 (33, 57); 13/18 patients suffered a pneumothorax; 8/18 had pulmonary hypertension, and 5/18 had necrotizing enterocolitis (NEC) at some point during their NICU admission.

**Table 1:** Demographic differences in subjects with length z-score ≥  − 2 vs. <  − 2 at 90 days of life

	All	Length z-score ≥ − 2	Length z-score < − 2	*p* value
	*N* = 18	7 (38.9%)	11 (57.9%)	
Sex				1.00
Female	4 (22.2%)	2 (25%)	2 (18.2%)	
Male	14 (77.8%)	5 (71.4%)	9 (81.8%)	
Birth weight (g)	2505 (2252, 2845)	2338 (2075, 3115)	2505 (2282, 2845)	0.82
Birth length (cm)	46 (42.9, 48.8)	46 (45, 48.3)	43 (42.25, 48)	0.41
Gestational age (wks)	35.85 (34.5, 37)	34.4 (34.1, 35.9)	36.1 (34.7, 37)	0.19
Premature (< 37 GA)	12 (66.7%)	6 (85.7%)	6 (54.5%)	0.17
Comorbidities				
Pneumothorax	13 (72.2%)	4 (57.1%)	9 (81.8%)	0.51
Pulmonary hypertension	8 (44.4%)	3 (42.9%)	6 (54.5%)	1.00
Necrotizing enterocolitis	5 (27.8%)	4 (42.9%)	1 (9.1%)	0.19
Required ECMO	5 (27.8%)	2 (28.5%)	3 (27.3%)	1.00
Days on ventilator	48 (33, 57)	43 (19.5, 54)	51 (44, 57)	0.26
Dialysis data				
Days at CKRT start	5 (4, 6)	4 (2, 4)	6 (5, 6)	0.04
Days on CKRT	108 (82, 154)	120 (95, 183)	98 (85, 142.5)	0.53
Transition to PD	11 (61.1%)	5 (71.4%)	7 (63.6%)	1.00
Age PD start (days)	89 (64.3, 127.5)	68 (41, 164)	89 (84, 119)	0.68
Dosing on CKRT				1.00
Era 1 (2000/1.73/m^2^/h)	5 (27.8%)	2 (28.5%)	3 (27.3%)	
Era 2 (24 ml/kg/h)	13 (72.2%)	5 (71.4%)	8 (72.7%)	
Nutritional goals				< 0.02
Era 1	11 (61.1%)	4 (36%)	7 (64%)	
Era 2	7 (38.9%)	7 (100%)	0 (0%)	
Prescribed calories*	106.8 (104.4–110.7)	118.2 (110–127)	105 (103.6–109.7)	0.12
Prescribed protein*	3.8 (3.5–4.0)	3.9 (3.7–4.0)	3.6 (3.5–4.0)	0.30
Alive at discharge	13 (72.2%)	6 (85.7%)	7 (63.64%)	0.60
Age at discharge/death	188 (113, 209.5)	188 (151.5, 212)	192 (110, 209.5)	0.59

^*^Average prescribed calories and protein based on 9 snapshots at days 10, 20, 30, 40, 50, 60, 70, 80, and 90

**Table 2:** Demographics differences between subjects with weight z-score ≥  − 2 vs. <  − 2 at 90 days of life

	All	Weight z-score ≥ − 2	Weight z-score < − 2	*p* value
	*N* = 18	10 (55.5%)	8 (42.1%)	
Sex				0.09
Female	4 (22.2%)	4 (40.0%)	0 (0%)	
Male	14 (77.8%)	6 (60.0%)	8 (100%)	
Birth weight (g)	2505 (2252, 2845)	2645 (2355, 3065)	2304 (2186, 2591)	0.33
Birth length (cm)	46 (42.9, 48.8)	46 (42.9, 48.9)	44.5 (42.75, 46.25)	0.53
Gestational age (weeks)	35.85 (34.5, 37)	35.8 (34.3, 37.8)	34.6 (35.2, 37)	0.76
Premature (< 37 weeks)	12 (66.7%)	7 (70.0%)	5 (62.5%)	1.00
Comorbidities				
Pneumothorax	13 (72.2%)	7 (70.0%)	8 (100%)	1.00
Pulmonary hypertension	8 (44.4%)	5 (50.0%)	4 (50%)	1.00
Necrotizing enterocolitis	5 (27.8%)	2 (20.0%)	3 (37.5%)	
Required ECMO	5 (27.8%)	3 (30.0%)	2 (25%)	1.00
Days on ventilator	48 (33, 57)	48 (30.3, 57.8)	51.5 (42.3, 57)	0.97
Dialysis data				
Day of CKRT start	5 (4, 6)	4.5 (4, 6)	5.5 (4, 6.25)	0.50
Days on CKRT	108 (82, 154)	126 (88.5, 181.0)	93.5 (80, 150)	0.37
Transition to PD	11 (61.1%)	8 (80.0%)	4 (50%)	0.23
PD start age (days)	89 (64.3, 127.5)	84 (61.3, 127.5)	102.5 (80, 128)	0.67
Dosing on CKRT				0.11
Era 1 (2000/1.73/m^2^/h)	5 (27.8%)	1 (10.0%)	4 (50%)	
Era 2 (24 ml/kg/h)	13 (72.2%)	9 (90.0%)	4 (50%)	
Nutrition era goals				< 0.01
First	11 (61.1%)	3 (27.7%)	8 (72.7%)	
Second	7 (38.8%)	7 (100.0%)	0 (0%)	
Prescribed calories*	106.8 (104.4–110.7)	109.9 (104.4–114.8)	105.4 (102.4–109.7)	0.2
Prescribed protein*	3.8 (3.5–4.0)	4.0 (3.8–4.1)	3.6 (3.4–3.7)	< 0.01
Alive at discharge	13 (72.2%)	9 (90.0%)	4 (50%)	0.12
Age at discharge/death	188 (113, 209.5)	194 (134.0, 217.5)	170 (95, 209.8)	0.33

^*^Prescribed calories and protein based on median of 9 snapshots at days 10, 20, 30, 40, 50, 60, 70, 80, and 90

### Overall outcomes

Of the 59 neonates who received CKRT at COA, 24 received long-term CKRT starting in the first 10 postnatal days, and 18/24 (75%) survived to 90 days. Of the 18 who survived to 90 days, 5 died prior to hospital discharge (101 (93, 217)). Of the 13 who survived to NICU discharge, 11/13 (85%) were discharged home with nightly peritoneal dialysis, one to in-center hemodialysis, and one regained some kidney function and was discharged home with CKD management.

All patients had negative changes in z-scores for both weight and length from 0 to 90 days. Median length z-score at birth was − 0.24 (− 1.1, 0.5), at 90 days − 2.66 (− 3.02, − 1.72), and with median change in score at 90 days − 2.1 (− 2.7, − 1.56). Median weight z-score at birth was 0.45 (− 0.28, 1.18), at 90 days − 1.54 (− 3.26, − 1.1) with delta at 90 days 2.41 (− 3.02, − 1.99).

### Evaluation of risk factors and outcomes

Of the 18 neonates who survived, 7/18 (38.9%) had a length z-score ≥  − 2 at 90 days. Table 1 shows the difference in demographics, comorbidities, and interventions between those with length z-score ≥  − 2 vs. <  − 2. Those with length z-score ≥  − 2 started CKRT earlier than those with length z-score <  − 2 (median (IQR) = 4 (2–4) vs. 6 (5–6); *p* = 0.04). No statistically significant differences were seen for patients who were in the CKRT Dose Era 1 vs. Dose Era 2. We did find that those in CKRT Nutrition Era 2 (at least 130 kcal/kg/day and at least 4 g/kg day) were more likely to have length z-score ≥  − 2 (7/7 (100%) vs. 3/11 (36%); *p* < 0.01). No other statistically significant differences were seen in the risk factors between groups.

Of the 18 neonates who survived, 10/18 (55.5%) had a weight z-score ≥  − 2 at 90 days. Table 2 shows the difference in demographics, comorbidities, and interventions between those with weight z-score ≥  − 2 vs. <  − 2. All four females had a weight z-score ≥  − 2 (*p* = 0.09). No statistically significant differences were seen for patients who were in the dosing of CKRT Era 1 vs. 2. Those in CKRT Nutrition Era 2 (at least 130 kcal/kg/day and at least 4 g/kg day) had higher weight z-score ≥  − 2 (7/7 (100%) vs. 3/11 (27.2%); *p* < 0.01). The average amount of prescribed protein was higher in those with higher weight z-score ≥  − 2 (4.0 (3.8–4.1) vs. 3.6 (3.4–3.7); *p* < 0.01), not controlling for dialysis modality. No other statistically significant difference was seen in the risk factors between groups.

### Prescribed calories and protein in Nutrition Eras 1 and 2

**Fig. 1:**
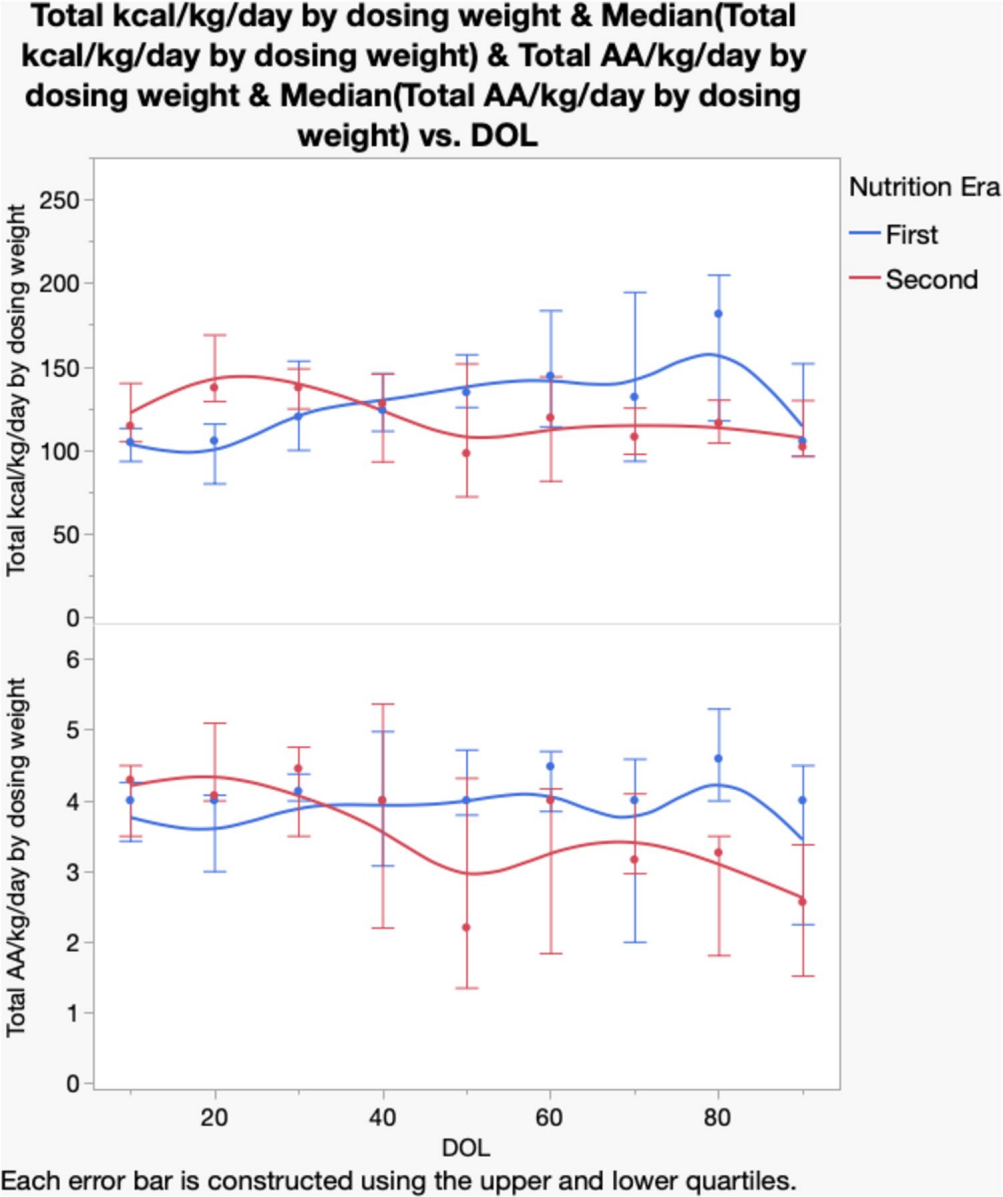
Calories and protein by day of life for Nutrition Eras

Figure 1 shows the prescribed calories and protein in subjects in Nutrition Era 1 vs. Era 2 on days 10, 20, 30, 40, 50, 60, 70, 80, and 90. In Era 1, the median calories and protein prescribed were lower than what was recommended (90–110 kcal/kg/day and 3.5 g/kg/day AA) at days 10, 20, and 30, but higher on days 50, 60, 70, 80, and 90. In Era 2, calorie and protein prescriptions were higher to start at the initiation of nutrition and reached recommended levels at 15 days; then, they started a downward trend around 30 days for both protein and energy. None of the data captures actual calories and protein consumed.

Further analysis was made into the demographics between the two Nutrition Eras (Table 3). There was no statistical significance among sex, weight, length, and gestational age. Those in Nutrition Era 1 had more days on the ventilator than those in Nutrition Era 2 (57 (45.5, 62) vs. 42 (26, 48); *p* = 0.05). In Nutrition Era 1, 5/11 (45.5%) transitioned to PD, as opposed to 7/7 (100%); *p* = 0.04) in Nutrition Era 2. Also to note, the PD start time was longer in Nutrition Era 1 (116, (89, 164) vs. 79 (44, 105); *p* = 22), exposing those patients to > 1 month of CKRT than Nutrition Era 2.

**Table 3:** Characteristics of patients by Nutrition Era

	All	Nutrition Era 1 90–110 kcal/kg/day 3.5 g AA/kg/day	Nutrition Era 2 At least 130 kcal/kg/day At least 4 kcal/kg/day	*p* value
	*N* = 18	*N* = 11 (61.1%)	*N* = 7 (38.9%)	
Sex				0.25
Female	4 (22.2%)	1 (9.1%)	3 (42.9%)	
Male	14 (77.8%)	10 (90.9%)	4 (57.1%)	
Birth weight (g)	2505 (2252, 2845)	2340 (2137, 2645)	2840 (2380, 3315)	0.18
Birth length (cm)	46 (42.9, 48.8)	44 (42.5, 46.5)	48.5 (43.3, 49.9)	0.30
Gestational age (wks)	35.85 (34.5, 37)	35.9 (34.6, 36.6)	35.7 (34.4, 37.1)	0.93
Premature (< 37 GA)	12 (66.7%)	9 (81.8%)	4 (57.1%)	1.00
Comorbidities				
Pneumothorax	13 (72.2%)	9 (81.8%)	4 (57.1%)	0.33
Pulmonary hypertension	8 (44.4%)	7 (63.6%)	1 (14.3%)	0.34
Necrotizing enterocolitis	5 (27.8%)	5 (45.5%)	0 (0%)	0.10
Required ECMO	5 (27.8%)	3 (27.3%)	2 (28.6%)	1.00
Days on ventilator	48 (33, 57)	57 (45.5, 63)	43 (26, 48)	0.05
Dialysis data				
Days at CKRT start	5 (4, 6)	5 (4, 6)	5 (3, 6)	0.75
Days on CKRT	108 (82, 154)	146 (88.5, 183)	108 (82, 126)	0.28
Transition to PD	11 (61.1%)	5 (45.5%)	7 (100%)	0.04
Age PD start (days)	89 (64.3, 127.5)	116 (89, 164)	79 (55, 105)	0.22
Dosing on CKRT				
Lower clearance rate Era 2 (24 ml/kg/h)	13 (72.2%)	6 (54.5%)	7 (100%)	0.10
Alive at discharge	13 (72.2%)	6 (54.5%)	7 (100%)	0.10
Age at discharge/death	188 (113, 209.5)	207 (125, 214)	155 (120, 194)	0.53

### Discussion

In this study, we show that many neonates who require CKRT do not grow well. We also show that the difference in recommended vs. prescribed is quite variable (Fig. 1). Additionally, we have not captured data on actual calories and protein consumed, and for this population, which can vary from what is prescribed. Intolerance, fluid restriction when CKRT is not running well, and titration between parenteral and enteral can all affect consumption of prescribed nutrition. Patients could have received more than prescribed nutrition if feed volume prescribed in the EMR was ordered to be titrated over the course of the day. Similarly, a patient could have received less than the prescribed amount if the prescribed feeding regimen was paused for intolerance, for a procedure requiring the patient to be NPO, or if the patient had complications with delivery of CKRT, such as access issues, which required time off circuit and fluid restriction. Although we have clear guidelines at our institution for where to start regarding energy and protein in neonates requiring CKRT, we were not able to feasibly or clearly capture the variation in nutrition prescriptions given for these patients throughout the study period.

We did see that calorie and protein recommendations were modifiable risk factors associated with growth. Specifically, we found that the Nutrition Era 2 (recommended kcal goal of at least 130 kcal/kg/day and amino acids of at least 4 g/kg/day to start at initiation of nutrition) was associated with lower rates of low weight and length z-scores compared to those in Nutrition Era 1, with lower calorie and protein recommendations. In this small sample, we were not able to decipher any other demographic parameters, procedures, or co-morbidities associated with poor growth, although we note that they were very common in our population.

Interestingly, we show that in Nutrition Era 1, the dose of calories and protein prescribed significantly increased over the first months after birth, while in the second era, these decreased. Some plausible explanations for these findings are that early nutrition is beneficial in establishing better growth. We found that in Era 1, it took > 30 days to reach recommended kcal and AA targets, compared with Era 2, where it only took 15 and 10 days to reach kcal and AA targets, respectively. Also, in general, infants respond to enteral and parenteral nutrition differently; they respond differently to types of supplementation, as well as having different patient factors. An additional plausible explanation is that we started at lower doses, and poor growth pushed the clinical team to provide more nutritional support. This, compared to Nutrition Era 2, demonstrated that our consistently higher goals may have met growth targets sooner. Another factor to consider is the PD start day for the two eras. For Nutrition Era 2, the median PD start day was a month earlier (79 days (55, 1055), compared to 116 days (89, 164); *p* = 0.22)) in Nutrition Era 1. Because our calorie and protein goals drop between the two modalities, this could be a reason we saw the lower prescriptions featured in Fig. 1 for Era 2.

Comparison data between the nutrition era groups shows that transitioning to PD is an indicator for better growth. Those who have higher calorie and protein goals were all able to start on PD, which is one step closer to discharge. The data also show that all of those in the second nutrition era survived to discharge. Due to the small sample size, it is hard to delineate whether patients who were on PD earlier grew better, or patients who were in the second era grew better and were able to transition to PD earlier and avoid complications associated with CKRT.

In addition, we show that daily weights are very possible in these subjects, even when they are on pressor and ventilator support. This is crucial in adequately identifying patient weight gain and modification of energy and protein targets, which could explain the change in those we see in Fig. 1. In fact, our program relies on twice daily weights for fluid and nutritional assessments, and our bedside RNs successfully capture these metrics on even the sickest babies. Figure 1 shows that we are still having a mismatch in calories and protein intake for weight that day vs. dosing weight. Identifying and updating our patient’s dosing weight more frequently would be impactful to more accurately prescribe the nutrition these patients need. Our practice has changed recently to updating dosing weights for neonates and infants 1–2 times per week to not miss out on nutrition and to better identify these patients’ growth patterns.

Nutrition studies in this population are limited. Many of the studies that mention neonates, CKRT, and nutrition just reiterate that CKRT allows for the delivery of nutrition but fails to address prescriptions appropriate for CKRT [11]. Furthermore, programs vary widely in the delivery of CKRT, including time and dose, which can impact information needed for a comparable review [3]. Assessment of the success of growth among this patient population varies among publications as well. We targeted calorie and protein prescriptions related to growth velocity, but other studies used assessment of total fluid intake to describe nutrition rather than capturing calorie data [8], making a comparison between institutions challenging. Finally, many studies have solidified the need for additional protein, trace minerals, and water-soluble vitamin supplementation, but have not researched additional calorie and specific protein needs [12]. The Pediatric Renal Nutrition Taskforce highlighted this issue as well, noting that there are different nutrition recommendations for patients with different etiologies of kidney disease, so even making standardized nutrition recommendations across all CKF is difficult [6].

The strength of this single-center analysis is that we captured a lot of demographic and nutritional data on the subject. This study represents data from our program where we offer a systematic approach to nutrition and CKRT clearance rates. The use of an extracorporeal therapy early after birth enables us to focus on the provision of nutrition in these patients without regard to fluid excess as we can achieve fluid homeostasis with CKRT. Despite this strength, we acknowledge that this was a small single-center study in a single program, which did not randomize patients to one regimen or another. We also recognize the possibility of survival bias as we removed any subjects who did not survive to the primary outcome’s timeframes of interest. We also recognize that the analysis of the amount of calories and protein by era was not done prospectively, and we did not capture the exact calories or protein consumed, just prescribed at that time point.

In conclusion, we show that although many patients who receive KRT in the neonatal period fail to adequately gain weight, the amount of calories and protein prescribed appears to be a modifiable risk factor for growth. Getting patients transitioned to peritoneal dialysis earlier appears to be an intervention that could increase growth for our patient population; however, there are multiple reasons for PD placement deferral or failure. Data still do not identify why patients transitioned to PD earlier grew or if patients grew so they could be transitioned successfully to PD. More studies are needed to determine what the optimal nutritional approaches to growth for infants on CKRT are, as this is a viable and increasingly used option for the critically ill.

Additionally, the creation of clinical practice guidelines by collaborative groups already in place such as WE-ROCK [13], the Neonatal Kidney Collaborative, and ICONIC Collaborative could be a huge benefit for this patient population in the future. In the meantime, we recommend that neonates are prescribed at least 130 kcal/kg/day and at least 4 g/kg/day of amino acids as soon as possible to start while on CKRT, with frequent assessment of growth by weight and BUN, and frequent modification of dosing weight.

## Supplementary Information

Below is the link to the electronic supplementary material.

ESM 1 Graphical abstract (PPT 260 KB)

ESM 2 DOCX (25.4 KB)

## Data Availability

The datasets generated during and/or analyzed during the current study are available from the corresponding author on reasonable request.
