# Supplementary material for: Growth in neonates with congenital kidney failure requiring continuous kidney replacement therapy
Source: Pediatr Nephrol. 2025 Aug 7;40(12):3733–41. doi: 10.1007/s00467-025-06887-y (PMC12549734; doi:10.1007/s00467-025-06887-y)
Supplement: Supplementary file 1 — Graphical abstract (PPT 260 KB) [file 467_2025_6887_MOESM1_ESM.ppt]

## Slide 1
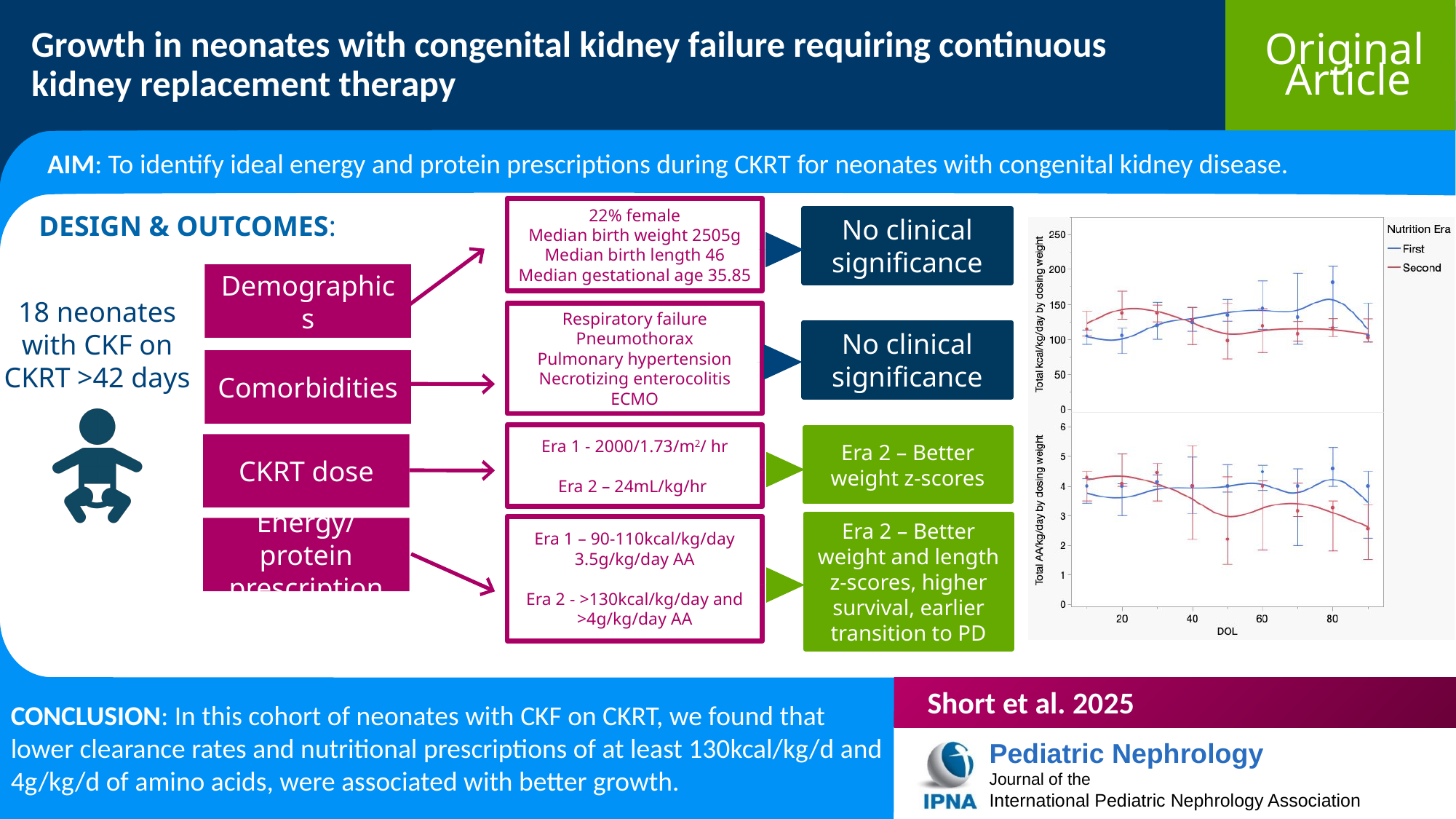

Growth in neonates with congenital kidney failure requiring continuous
kidney replacement therapy
AIM: To identify ideal energy and protein prescriptions during CKRT for neonates with congenital kidney disease.
22% female
Median birth weight 2505g
Median birth length 46
Median gestational age 35.85
DESIGN & OUTCOMES:
No clinical significance
Demographics
18 neonates with CKF on CKRT >42 days
Respiratory failure
Pneumothorax
Pulmonary hypertension
Necrotizing enterocolitis
ECMO
No clinical significance
Comorbidities
Era 1 - 2000/1.73/m2/ hr
Era 2 – 24mL/kg/hr
Era 2 – Better weight z-scores
CKRT dose
Era 2 – Better weight and length z-scores, higher survival, earlier transition to PD
Era 1 – 90-110kcal/kg/day 3.5g/kg/day AA
Era 2 - >130kcal/kg/day and >4g/kg/day AA
Energy/protein prescription
Short et al. 2025
CONCLUSION: In this cohort of neonates with CKF on CKRT, we found that lower clearance rates and nutritional prescriptions of at least 130kcal/kg/d and 4g/kg/d of amino acids, were associated with better growth.
