# Supplementary material for: Growth in neonates with congenital kidney failure requiring continuous kidney replacement therapy
Source: Pediatr Nephrol. 2025 Aug 7;40(12):3733–41. doi: 10.1007/s00467-025-06887-y (PMC12549734; doi:10.1007/s00467-025-06887-y)
Supplement: Supplementary file 2 — DOCX (25.4 KB) [file 467_2025_6887_MOESM2_ESM.docx]

Supplement Table 1: Comorbidities and surgeries (did not include hemodialysis, peritoneal dialysis catheter, or gastric tube placement)

| Patient number | Comorbidities | Surgeries/complications | Bacteremia or urosepsis |
| --- | --- | --- | --- |
| 1 | - Premature infant - Cardiac tamponade with emergent pericardiocentesis - Pulmonary hypoplasia - Pulmonary hypertension - Pneumothorax - Tracheomalacia - Necrotizing enterocolitis | - Colostomy - Vesicostomy - Urethral dilation with bladder stone removal | Y |
| 2 | - Premature infant - Necrotizing enterocolitis - Pulmonary hypoplasia - Pneumothorax - Pulmonary hypertension | - Ostomy and mucous fistula - Ostomy takedown - ECMO cannulation | Y |
| 3 | - Premature infant - Pulmonary hypoplasia - Pneumothorax |  | Y |
| 4 | - Premature infant - Pulmonary hypoplasia - Pneumothoraces - Pulmonary hypertension - Focal seizures |  | Y |
| 5 | - Pulmonary hypoplasia - Pneumothoraces |  | Y |
| 6 | - Premature infant - Pulmonary hypoplasia - Pneumothoraces - Pulmonary hypertension - Nectrotizing enterocolitis - VACTERL - Imperforate anus | - Ostomy and mucous fistula | Y |
| 7 | - Premature infant - Pulmonary hypoplasia - Pneumothorax - Pulmonary hypertension - Trisomy 21 - Imperforate anus - Intravenous hemorrhage - Seizure disorder | - Sigmoid ostomy | Y |
| 8 | - Pulmonary hypoplasia - Pneumothoraces - Pulmonary hypertension - Necrotizing enterocolitis | - Posterior urethral valve (PUV) ablation | Y |
| 9 | - Pulmonary hypoplasia - Pneumothorax - Pulmonary hypertension | - ECMO cannulation - ECMO decannulation - Posterior urethral valve ablation - Bilateral inguinal hernia repair | Y |
| 10 | - Premature infant - Pulmonary hypoplasia - Pneumothorax - Pulmonary hypertension - Urogenital/anorectal abnormalities - Stomach perforation - Necrotizing enterocolitis | - ECMO cannulation - ECMO decannulation - Bowel resection |  |
| 11 | - Premature infant - Pulmonary hypoplasia - Pneumothoraces | - Bilateral nephrostomy tubes - Bilateral ureterostomies - Bilateral ureteral stents | Y |
| 12 | - Pulmonary hypoplasia - Pulmonary hypertension | - ECMO cannulation - ECMO decannulation - Bilateral nephrectomy | Y |
| 13 | - Premature infant - Pulmonary hypoplasia - Patent urachus | - Excision of patent urachus |  |
| 14 | - None |  |  |
| 15 | - Premature infant - Pulmonary hypoplasia - Pneumothorax - Pulmonary hypertension - Seizure disorder | - PUV ablation - Wound debridement |  |
| 16 | - Premature infant - Pulmonary hypoplasia - Pneumothorax - Pulmonary hypertension - Thrombocytopenia | - PUV ablation - Wound debridement |  |
| 17 | - Premature infant - Pulmonary hypoplasia - Pneumothorax - Pulmonary hypertension - Cerebrovascular accident - Heparin induced thrombocytopenia | - PUV ablation | Y |
| 18 | - Pulmonary hypoplasia - Pneumothorax - Pulmonary hypertension | - ECMO cannulation - ECMO decannulation - Bilateral nephrectomy | Y |
